# Aging and loss decision making: increased risk aversion and decreased use of maximizing information, with correlated rationality and value maximization

**DOI:** 10.3389/fnhum.2015.00280

**Published:** 2015-05-13

**Authors:** Yoanna A. Kurnianingsih, Sam K. Y. Sim, Michael W. L. Chee, O’Dhaniel A. Mullette-Gillman

**Affiliations:** ^1^Department of Psychology, Faculty of Arts and Sciences, National University of SingaporeSingapore, Singapore; ^2^Centre for Cognitive Neuroscience, Neuroscience and Behavioral Disorders Program, Duke-NUS Graduate Medical SchoolSingapore, Singapore; ^3^Centre for Ageing Studies, Temasek PolytechnicSingapore, Singapore; ^4^SINAPSE Institute for Cognitive Science and Neurotechnologies, National University of SingaporeSingapore, Singapore

**Keywords:** aging, decision making, risk, strategy, losses, gains, uncertainty, ambiguity

## Abstract

We investigated how adult aging specifically alters economic decision-making, focusing on examining alterations in uncertainty preferences (willingness to gamble) and choice strategies (what gamble information influences choices) within both the gains and losses domains. Within each domain, participants chose between certain monetary outcomes and gambles with uncertain outcomes. We examined preferences by quantifying how uncertainty modulates choice behavior as if altering the subjective valuation of gambles. We explored age-related preferences for two types of uncertainty, risk, and ambiguity. Additionally, we explored how aging may alter what information participants utilize to make their choices by comparing the relative utilization of maximizing and satisficing information types through a choice strategy metric. Maximizing information was the ratio of the expected value of the two options, while satisficing information was the probability of winning. We found age-related alterations of economic preferences within the losses domain, but no alterations within the gains domain. Older adults (OA; 61–80 years old) were significantly more uncertainty averse for both risky and ambiguous choices. OA also exhibited choice strategies with decreased use of maximizing information. Within OA, we found a significant correlation between risk preferences and choice strategy. This linkage between preferences and strategy appears to derive from a convergence to risk neutrality driven by greater use of the effortful maximizing strategy. As utility maximization and value maximization intersect at risk neutrality, this result suggests that OA are exhibiting a relationship between enhanced rationality and enhanced value maximization. While there was variability in economic decision-making measures within OA, these individual differences were unrelated to variability within examined measures of cognitive ability. Our results demonstrate that aging alters economic decision-making for losses through changes in both individual preferences and the strategies individuals employ.

## Introduction

Aging has been suggested to result in alterations in numerous cognitive processes, but it is unclear what specific alterations in economic decision making may take place. Understanding age-related alterations of economic decision-making is important, as elderly persons are often less financially resilient and often considered more likely to be targets of consumer fraud (Lee and Soberon-Ferrer, 1997; Castle et al., 2012; Ross et al., 2014). In this study, we specifically test whether economic decision making is altered in a healthy sample of older adults (OA), through tasks that control for dissociable processes (such as learning or memory effects).

At the most general cognitive levels, aging is associated with decreased processing speed (Salthouse, 2000) and deficits in a range of cognitive processes, including inhibition (Lustig et al., 2007), executive functions (Goh et al., 2012), episodic memory (Shing et al., 2008), and reward learning (Mell et al., 2005). These changes in cognitive abilities may in turn affect economic decision-making, such as the propensity to invest (Christelis et al., 2010; Korniotis and Kumar, 2011).

Prior studies utilizing decision making tasks have suggested alterations across a range of tasks, including the Iowa Gambling Task (IGT; Denburg et al., 2005, 2009; Wood et al., 2005; Fein et al., 2007; Zamarian et al., 2008; Baena et al., 2010; Carvalho et al., 2012), the Gambling Task (Kovalchik et al., 2005), Balloon Analogue Risk Task (BART; Henninger et al., 2010; Rolison et al., 2012), and the Cambridge Gambling Task (CGT; Deakin et al., 2004; Henninger et al., 2010). However, it is unclear whether such studies reflect specific alterations in economic decision making, as these tasks feature outcome resolution at the end of each trial. As aging has been found to impact reward learning (Mell et al., 2005; Eppinger et al., 2011), it is unclear if the observed behavioral changes are merely an extension of age-related decline in learning or if they truly reflect altered preferences or strategies (see Mata et al., 2011; Worthy et al., 2011). The former account is supported by some (Henninger et al., 2010; Boyle et al., 2011) but not other studies (Anderson et al., 2013).

Here, we examined how economic decision-making may be specifically altered in relatively healthy OA, focusing on two aspects of economic decision-making: uncertainty preferences (risk and ambiguity) and choice strategies.

Uncertainty preferences are a measure of how an individual responds to the unknown future resolution of a probabilistic option (i.e., a gamble). Uncertainty can be described as being of two types, as risk when the probabilities of possible outcomes are known or can be estimated, or as ambiguity when the probabilities of possible outcomes are not well defined (Knight, 1921; Ellsberg, 1961; Camerer and Weber, 1992).

Uncertainty preferences differ depending on whether individuals are facing potential gains or losses (Prospect Theory, Kahneman and Tversky, 1979). Given the ubiquity of losses in real-world decisions, it is important to understand how aging may differentially impact decision making across both the gains and losses domains. Across both the gains and losses domains, prior behavioral studies investigating age-related modulation of uncertainty preferences have resulted in inconsistent findings. In the gains domain, while some studies found OA to be more risk averse than younger adults (YA; Lauriola and Levin, 2001a; Albert and Duffy, 2012; Mather et al., 2012; Tymula et al., 2013), others did not show age-related effects (Mikels and Reed, 2009; Sproten et al., 2010). Inconsistencies have also been observed in the losses domain with some studies suggesting that OA are more risk averse (Mikels and Reed, 2009), and others suggesting that they are more risk seeking (Lauriola and Levin, 2001a; Mather et al., 2012). Only two studies have investigated age-related alterations of ambiguity preferences, with one suggesting that OA are less ambiguity averse than YA in the gains domain (Sproten et al., 2010) and the other finding no alterations (Tymula et al., 2013). Only one prior study has investigated age-related alteration of ambiguity preferences in the losses domain, finding OA were slightly more risk averse than YA. Neural evidence further suggests that we may anticipate an asymmetry in age-related modulation across the gains and losses domains. Samanez-Larkin et al. (2007) found reduced responsiveness in OA to anticipated monetary losses within striatal regions, while showing similar modulations to YA in the gains domain.

Beyond preferences, decision making is also dependent on the strategy one employs to utilize available information to reach their decision. For example, when choosing between two gamble options, one can simply consider the probability of winning for each option, or one can calculate and compare the expected value of each. In a potentially related domain, previous studies have reported that OA tend to use simpler and less demanding strategies for decision making involving probabilities (Kim et al., 2005; Rafaely et al., 2006). However, no prior study has investigated age-related differences in strategy use in monetary decision making.

In the present study, we examined how aging effects uncertainty preferences and choice strategies by contrasting relatively healthy OA with YA. To evaluate age-related differences, participants engaged in two incentive-compatible decision tasks (one with gains and one with losses), from which we computed their uncertainty preferences (risk and ambiguity) and quantified the choice strategy they employed to reach their decisions. Our *a priori* hypotheses were that: (1) healthy aging would result in no alteration of uncertainty preference in the gains domain, (2) OA would be less risk- and ambiguity-seeking in the losses domain, and (3) OA would present diminished choice strategies across both the gains and losses domains.

## Materials and Methods

### Participants

Data for the YA group were collected from 62 undergraduate students studying at the National University of Singapore (NUS; 24 males; age range = 19–26 years, age mean ± SD = 21.90 ± 1.69 years). Data for the OA group were collected from 39 cognitively healthy participants of the Singapore Longitudinal Brain Aging Study (Chee et al., 2009). These participants were screened, to exclude any of the following: (1) history of significant vascular events (i.e., myocardial infarction, stroke, or peripheral vascular disease), (2) history of malignant neoplasia of any form, (3) history of cardiac, lung, liver, or kidney failure, (4) active or inadequately treated thyroid disease, (5) active neurological or psychiatric conditions, (6) a history of head trauma with loss of consciousness, (7) a Mini-Mental State Examination (MMSE; Folstein et al., 1975) score <26, (8) a 15-point modified-Geriatric Depression Screening Scale (GDS; Sheikh and Yesavage, 1986), or (9) a history of illicit substance use.

All participants provided informed consent under a protocol approved by the NUS Institutional Review Board.

Two OA were excluded from analyses due to gross task performance issues in the monetary decision tasks, resulting in a final sample of 37 OA (22 females; age range of 61–80 years, mean ± SD = 68.66 ± 5.15 years). The demographics of the final sample of YA and OA participants are listed in **Table 1**. During their sessions, participants also performed additional behavioral tasks and surveys unrelated to this study.

**Table 1:** Participant demographics.

Younger adults (YA)	*N* = 62
Female, %	61.29
Age, years	22 ± 1.7

**Older adults (OA)**	***N* = 37**

Female, %	56.76
Age, years	69 ± 5.5
Education, years	12.1 ± 3.4
MMSE	28.1 ± 1.4
GDS	0.97 ± 1.38

MMSE, Mini mental state examination; GDS, Geriatric depression screening.

### Experimental Design

Data was collected as part of a larger-ongoing study. For the measures included in this report, participants underwent multiple measures of cognitive ability and performed two monetary decision making tasks (the first for the gains domain and the second for the losses domain).

#### Measuring Cognitive Ability in OA

Cognitive ability in OA was evaluated across five domains: (1) attention and working memory, (2) verbal memory, (3) visuospatial memory, (4) executive functioning, and (5) processing speed. Attention and working memory was assessed with the Digit Span (Wechsler, 1997) and a computerized version of a Spatial Span task. Verbal memory was evaluated using Rey Auditory Verbal Learning Test (RAVLT; Lezak et al., 2004). Visuospatial memory was evaluated using a Visual Paired Associate test. Executive functioning was evaluated using a Categorical Verbal Fluency test (using categories of animals, vegetables, and fruits), the Design Fluency test (Delis et al., 2001), and the Trail Making Test (TMT) B (Reitan and Wolfson, 1985). Processing speed was assessed with the TMT A (Reitan and Wolfson, 1985) and the Symbol–Digit Modalities Test (SDMT; Smith, 1991). To limit the number of comparisons, individual test scores were standardized (*z*-transformation) and combined within each categorical domain. We examined whether these cognitive domains are related to economic measures by correlating the composite scores from each of the five cognitive domains with our uncertainty preference and choice strategy metrics. The significance of these correlations was adjusted using Bonferroni correction for multiple comparisons with a threshold of *p* < 0.01 (i.e., correcting for the five cognitive domains).

#### Uncertainty Preference Tasks

**FIGURE 1:**
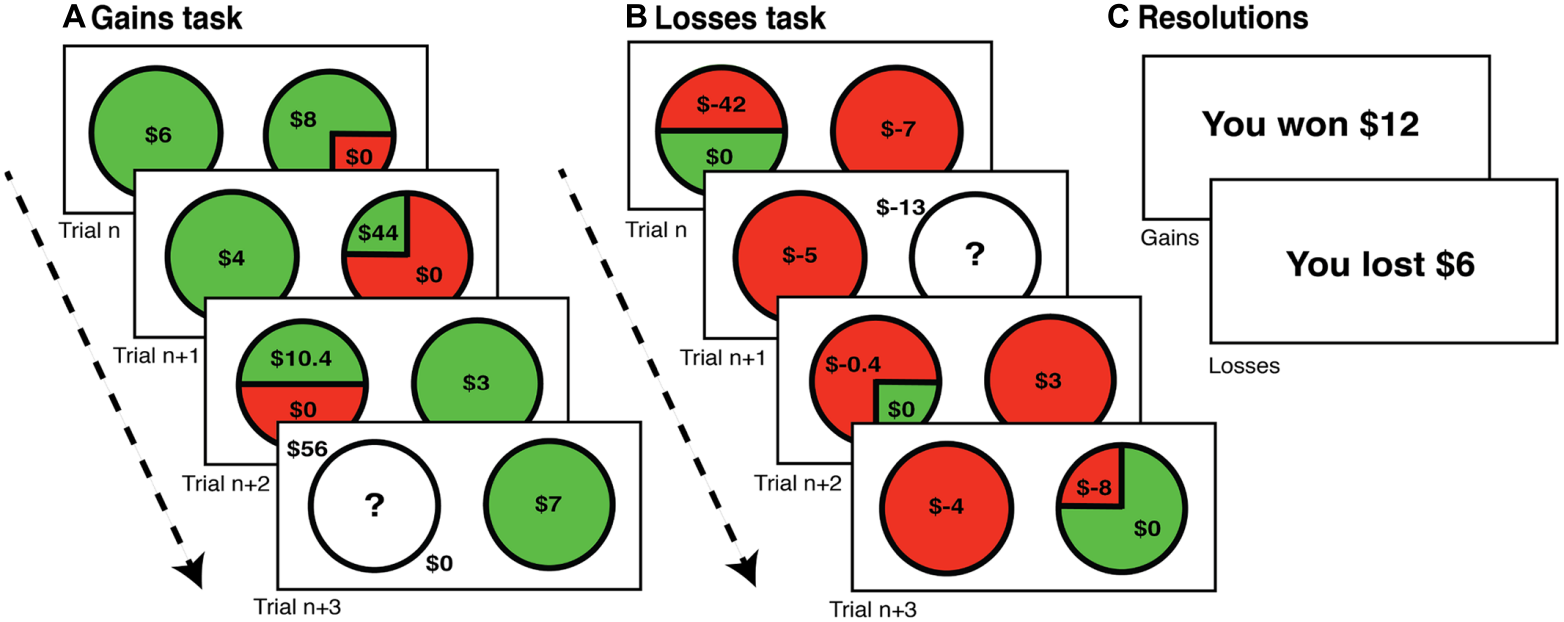
**Task timelines.** Participants performed two monetary decision-making tasks. One in the **(A)** gains domain (rewards) followed by a **(B)** losses domain version. In each trial, participants were asked to choose between a certain or a gamble option, with unconstrained response time. **(C)** Participants’ payments were based on random selection and resolution of one trial from each task, selected and resolved at the end of the entire experiment.

Uncertainty preferences (risk and ambiguity) were gathered through two monetary decision making tasks (see **Figure 1**), with each task oriented toward either the gains or losses domains. All participants performed the uncertainty-gains task followed by the uncertainty-losses task. On each trial of each task, participants chose between a certain option and a gamble option. Participants were informed that reimbursement would be determined at the end of the experiment based on random selection and resolution of one trial from each task. No resolutions were provided before the end of the entire experiment to eliminate alterations of preferences and choice strategies due to inter-trial learning from trial outcomes. Data collection and analyses were achieved using MATLAB (Mathworks, Natick, MA, USA) with Psychophysics Toolbox (Brainard, 1997) for trial presentation.

The uncertainty-gains task (Stanton et al., 2011), consisted of 165 trials, in which the participant chose between a certain option and a gamble option, which was either risky or ambiguous. For both gamble types, losses always resulted in $0 outcome. For risky gambles, there were five certain options ($3, $4, $5, $6, and $7), three probabilities of winning (25, 50, and 75%) and the value of the potential win ranged from $2 to $98, dependent upon the ratio of the expected value of the gamble to the certain option [relative expected value (rEV) or EV_G_/V_c_] for that trial. The trial matrix was constructed based on examining nine different rEVs (0.5, 1.0, 1.3, 1.6, 1.9, 2.2, 2.5, 3.0, and 3.5). With three probabilities of winning and the five different certain values, there were 15 trials for each level of rEV. For ambiguous gambles, six rEVs were examined (0.5, 1.0, 2.0, 3.0, 4.0, and 6.0), calculated using an assumed 50% probability of winning (by the law of large numbers). This resulted in five trials at each rEV, given the five values of the certain option.

The uncertainty-losses task consisted of 200 trials, closely mirroring the uncertainty-gains task, save for shifting the valence and adjusting the rEV values to allow for an anticipated increase in risk-seeking preferences (Kahneman and Tversky, 1979). There were five certain loss options (-$3, -$4, -$5, -$6, and -$7) with 10 examined rEVs (0.1, 0.3, 0.5, 0.8, 1.0, 1.3, 1.5, 2.0, 3.0, and 4.0); this adjusted range resulted in potential gamble losses ranging from -$0.4 to -$112. With three probabilities of winning (25, 50, and 75%) and the five different certain values, there were 15 trials for each level of rEV, as in the gains domain. These 10 rEV values were also examined for ambiguous gambles, calculated using an assumed 50% probability of winning. This resulted in five ambiguous trials at each rEV, given the five values of the certain option.

### Quantifying Uncertainty Preferences

Within each task, we quantified risk and ambiguity preferences by utilizing individual’s choice functions to find the ratio of the expected values of the gamble to the certain option at which participants were indifferent between the two. Each preference value is an expression of the degree and direction in which the participant’s choice behavior suggests they are modulating the subjective expected value of the gamble due to the outcome being unknown.

**FIGURE 2:**
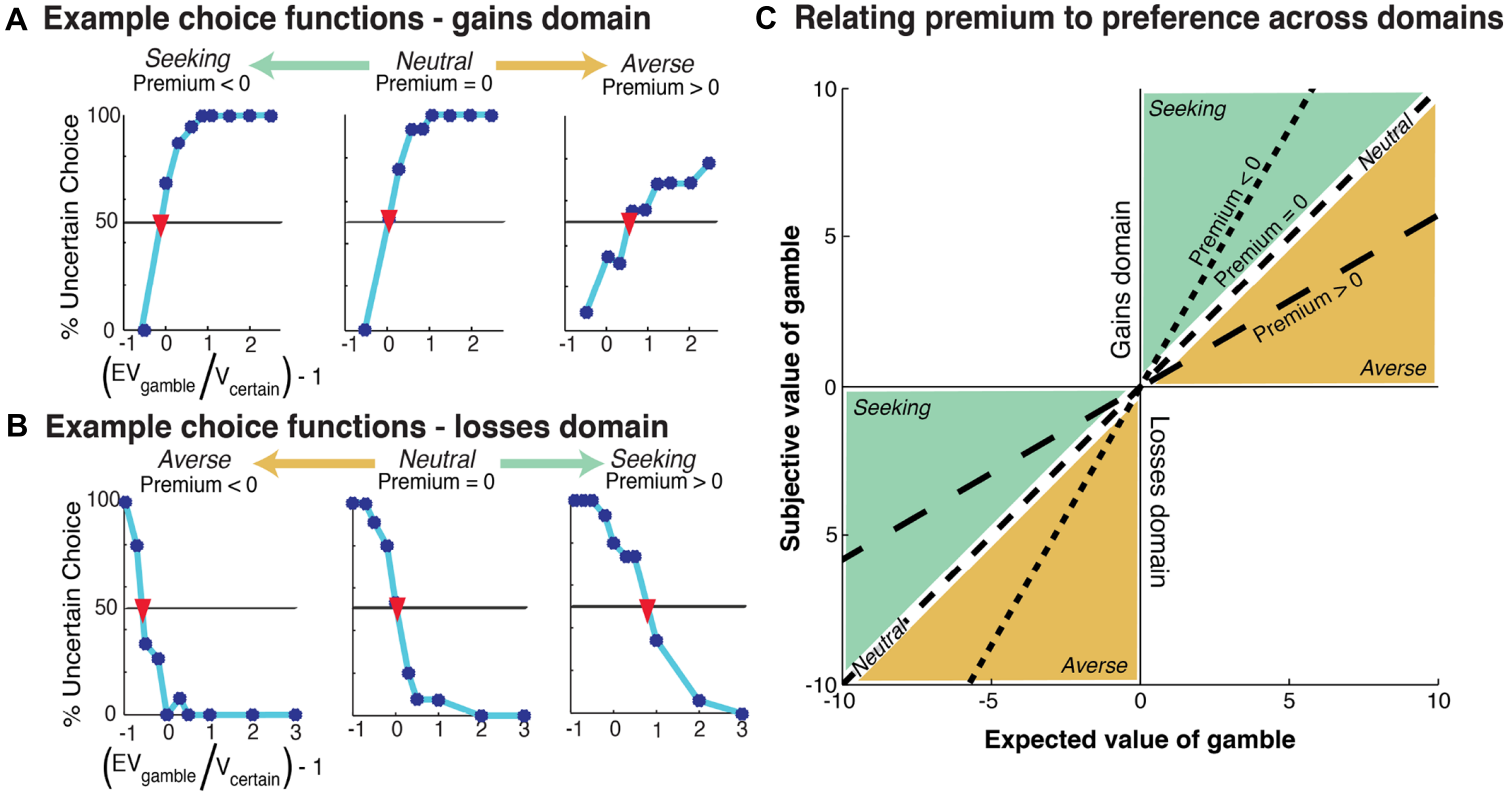
**Example participant choice functions. (A)** Gains domain, the range of risk preferences across participants is represented from risk seeking (left) to risk averse (right). The indifference point of each choice function is shown with a red inverted-triangle. Risk premium is defined as the value on the ‘(EV_G_/V_c_) - 1’ (*x*-axis) at this indifferent point. **(B)** Losses domain, the range of risk preferences is represented from risk averse (left) to risk seeking (right). **(C)** Relationship between premium metric and risk preference. Premium value corresponds to the slope of the line. Note that, as the premium value modulates the absolute expected value of the gamble, its relationship to preference (averse or seeking) is inverted between the gains and losses domains – e.g., positive premium values reflect risk-averse preferences in the gains domain and risk-seeking in the losses domain.

For each participant, four preference values were calculated (risk and ambiguity for the gains and losses domains) through psychometric indifference point analyses (Stanton et al., 2011). For each, a choice function was constructed based on the proportion of gamble options selected at each rEV. Examples of choice functions for individual participants within the gains domain are shown in **Figure 2A** and for the losses domains in **Figure 2B**. The indifference point was defined as the first point at which the projected choice function crossed 50%. We subtracted 1 from this indifference value to generate a ‘premium’ value. As such, the premium measures the degree to which the participant subjectively modifies the absolute expected value of a gamble due to outcome uncertainty. A zero premium reflects no change, a positive premium shows diminished valuation, and a negative premium indicates enhanced valuation. These calculations were performed separately for risk and ambiguity in each domain, gains, and losses, resulting in four independent premium values.

On a technical note, our quantification of uncertainty preferences assumes a linear relationship between value and utility across the range of possible outcomes (∼$100 in each task). While non-linearities may be evident when dealing with much larger sums (i.e., the difference in marginal utility for a dollar when you have 50 or when you have 1 million), the required rate of diminishing marginal utility to produce non-negligible non-linearities within a $100 range would result in highly untenable preferences when dealing with any large economic choice (Rabin, 2000).

As the premium metric quantifies the relative alteration of the absolute expected value of the gamble, its relation to preference (aversion and seeking) is inverted over the gains and losses domains (see **Figure 2C**). A positive premium in the gains domain indicates diminished absolute valuation of the gamble, which is also diminished valuation relative to the certain option. In the losses domain the same positive premium value still indicates diminished absolute valuation of the gamble, however, this is a relative *increase* in valuation compared to the certain option as the expected value of the gamble becomes less negative. As such, the interpretation of premium values into preference requires a reversal across domains (see **Figure 2C**). Therefore, in the gains domain, positive premium values show aversion and negative premium values indicate seeking, while in the losses domain, positive premium values indicate seeking and negative premium values indicate aversion. Neutrality corresponds to zero premium values in both domains.

We note that in a prior study using the uncertainty-gains task in a larger sample (N∼300, Stanton et al., 2011), we found that our psychometric premium values were highly correlated (correlations over | 0.6 |) with power function preference values (Prelec, 1998). We note now, similar high correlations between these measures of risk preference within the losses domain [Risk losses *r*(93) = -0.71, *p* < 0.0001; Ambiguity losses *r*(92) = -0.765, *p* < 0.0001]. For empirical reasons, due to the specific design of this task, we prefer the psychometric premium metric over the power-function measure [for a full description of these reasons, please see Stanton et al. (2011), Supplemental].

A small number of participants had choice functions that did not cross the indifference point (50% acceptance of gamble), preventing the psychometric determination of their premium values. Our data cannot resolve whether such participants were simply not performing the task correctly or if such participants had extreme preferences (we cannot differentiate between a participant who employed a strict heuristic (such as ‘always choose the certain/gamble option’) from one that considered the options but always selected the certain/gamble option because they are truly that averse/seeking to the gamble). This resulted in the exclusion of variable numbers of participants across the uncertainty metrics and domains (risk gains: 10 OA and 10 YA; risk losses: 2 OA and 2 YA; ambiguity gains: 14 OA and 23 YA; and ambiguity losses: 1 OA and 3 YA). Importantly, there were no significant differences in the proportions of participants excluded across the OA and YA for any cell [risk gains: χ^2^ (1, *N* = 99) = 1.71, *p* = 0.19; risk losses: χ^2^ (1, N = 99) = 0.284, *p* = 0.59; ambiguity gains: χ^2^ (1, *N* = 99) = 0.005, *p* = 0.94; and ambiguity losses: χ^2^ (1, *N* = 99) = 0.27, *p* = 0.60].

### Quantifying Choice Strategy

We examined whether aging altered what information participants relied upon to make their decisions through the use of a choice strategy metric. For each participant, we performed four independent linear regressions, two for each domain. Each regression determined the influence of a specific informational factor on choice in risk trials. We examined two factors: (1) the rEV of the options, and (2) the probability of winning in the gamble option (pWIN). Importantly, our task designs fully orthogonalize the pWIN and rEV factors (i.e., in each task the correlation of the values of pWIN and rEV across trials is zero).

The *r*^2^-value derived from each regression is a direct expression of the maximal amount of an individual’s choice variance (across trials) that can be accounted for by the examined factor (for examples, see **Figures 4A–D**). We directly contrasted utilization of these two competing trial-information sources by subtracting the *r*-squares of the rEV and pWIN factors. This results in our *choice strategy* metric (see **Figures 4E–H**), which directly measures how much more each participants’ choice behavior can be explained by the cognitively demanding calculation of the rEV of the options than by simple utilization of the visually available probability of winning the gamble.

This choice strategy metric is positive when participants utilize the rEV information more, negative when they focus on the pWIN information, and zero when they use the two equally. For example, a participant whose decisions were solely based on the value of pWIN (e.g., accepting all gambles with a 75% chance of winning) would have a high pWIN *r*^2^-value, a low rEV *r*^2^-value, and therefore a highly negative choice strategy. Similarly, a participant whose choices were determined by comparing the expected values of the gambles would have a high *r*^2^-value for rEV and low pWIN, resulting in a positive choice strategy value. Participants were considered to be ‘maximizing’ when they used the rEV information more and ‘satisficing’ when they used the pWIN information more, as focusing on pWIN allows for decisions through extremely simple heuristics (‘how much of the gamble pie is green?’) requiring little cognitive effort, while utilization of the rEV information maximizes long-run outcomes but requires several layers of effortful cognitive calculation.

We note that we opted to focus on the rEV and pWIN factors due to task design. While rEV and pWIN are orthogonal, other trial factors do not share this feature. For example, in the gains task the absolute value of the possible win is highly correlated to both the rEV and pWIN factors [rEV: *r*(133) = 0.604, *p* < 0.0001; pWIN: *r*(133) = -0.576, *p* < 0.0001], with similar correlations in the losses task.

### Relationship between Risk Preference and Choice Strategy

As we found significant age-related effects for both uncertainty preferences and choice strategies within the losses domain, we looked for a possible interaction by examining the correlation between these metrics within each age group.

## Results

### Cognitively Intact Older Sample

Our OA participants were cognitively unimpaired (MMSE ≥ 26), exhibiting psychometric test scores comparable to healthy participants studied elsewhere (**Table 2**, comparing TMT A, SDMT from Hsieh and Tori, 2007; TMT A and B from Tombaugh, 2003; Digit Span from Hedden et al., 2002; RAVLT from Davis and Klebe, 2001).

**Table 2:** Cognitive measures in OA.

Cognitive domain	Psychometric test	Mean ± SD
**Attention and working memory**	Digit Span forward	10.0 ± 2.3
	Digit Span backward	7.2 ± 1.9
	Spatial Span forward	7.5 ± 1.5
	Spatial Span backward	6.9 ± 1.5
**Processing speed**	SDMT (written)	44.6 ± 10.0
	SDMT (oral)	51.0 ± 12.0
	TMT A (s)	40.5 ± 14.0
**Verbal memory**	RAVLT	
	Sums of trials 1–5	51.4 ± 7.5
	Immediate recall list A	4.8 ± 1.6
	Delayed recall list A	10.9 ± 2.4
	Recognition list A	14.1 ± 1.9
**Visuospatial memory**	Visual paired associates	
	Sums of trials 1–4	16.9 ± 5.8
	Delayed recall	5.1 ± 2.0
**Executive functioning**	Categorical fluency	43.2 ± 7.3
	Design fluency	27.1 ± 7.5
	TMT B (s)	92.2 ± 41.8

SDMT, Symbol digit modalities test; TMT, Trail making test; RAVLT, Rey auditory verbal learning test.

### Relationship between Economic Measures and Cognitive Ability in OA

To examine whether differences in cognitive ability within our OA sample may alter economic preferences, we examined the relationships between our economic metrics and cognitive ability within our OA sample. Cognitive ability was quantified across five cognitive domains – attention and working memory, verbal memory, visuospatial memory, executive functioning, and processing speed (**Table 3**). To compare each of these five domains to each economic metric, we set a Bonferroni corrected significance threshold of *p* < 0.01 (correcting for the five examined cognitive domains), followed strictly as this was an ancillary component of the study. No significant correlations were found between performance on these cognitive domains and our uncertainty preferences (risk or ambiguity) or choice strategies.

**Table 3:** Relationships between decision making metrics and cognitive performance in OA.

Cognitive domain	Gains		Losses	
	Premium	Strategy	Premium	Strategy
Attention and working memory	*r*(25) = -0.03 *p* = 0.94	*r*(31) = 0.28 *p* = 0.12	*r*(33) = 0.43 *p* = 0.010	*r*(35) = 0.02 *p* = 0.89
Verbal memory	*r*(25) = -0.10 *p* = 0.62	*r*(31) = -0.20 *p* = 0.27	*r*(33) = 0.08 *p* = 0.64	*r*(35) = -0.08 *p* = 0.63
Visuospatial memory	*r*(25) = -0.06 *p* = 0.77	*r*(31) = 0.01 *p* = 0.95	*r*(33) = 0.29 *p* = 0.10	*r*(35) = -0.14 *p* = 0.40
Executive functioning	*r*(25) = -0.01 *p* = 0.95	*r*(31) = 0.13 *p* = 0.47	*r*(33) = -0.18 *p* = 0.31	*r*(35) = 0.14 *p* = 0.41
Processing speed	*r*(25) = -0.09 *p* = 0.65	*r*(31) = 0.18 *p* = 0.32	*r*(33) = 0.28 *p* = 0.11	*r*(35) = 0.22 *p* = 0.20

To account for multiple comparisons across the five cognitive domains, a Bonferroni corrected significance threshold of *p* < 0.01 was applied.

### Effects of Aging on Risk and Ambiguity Preferences

**FIGURE 3:**
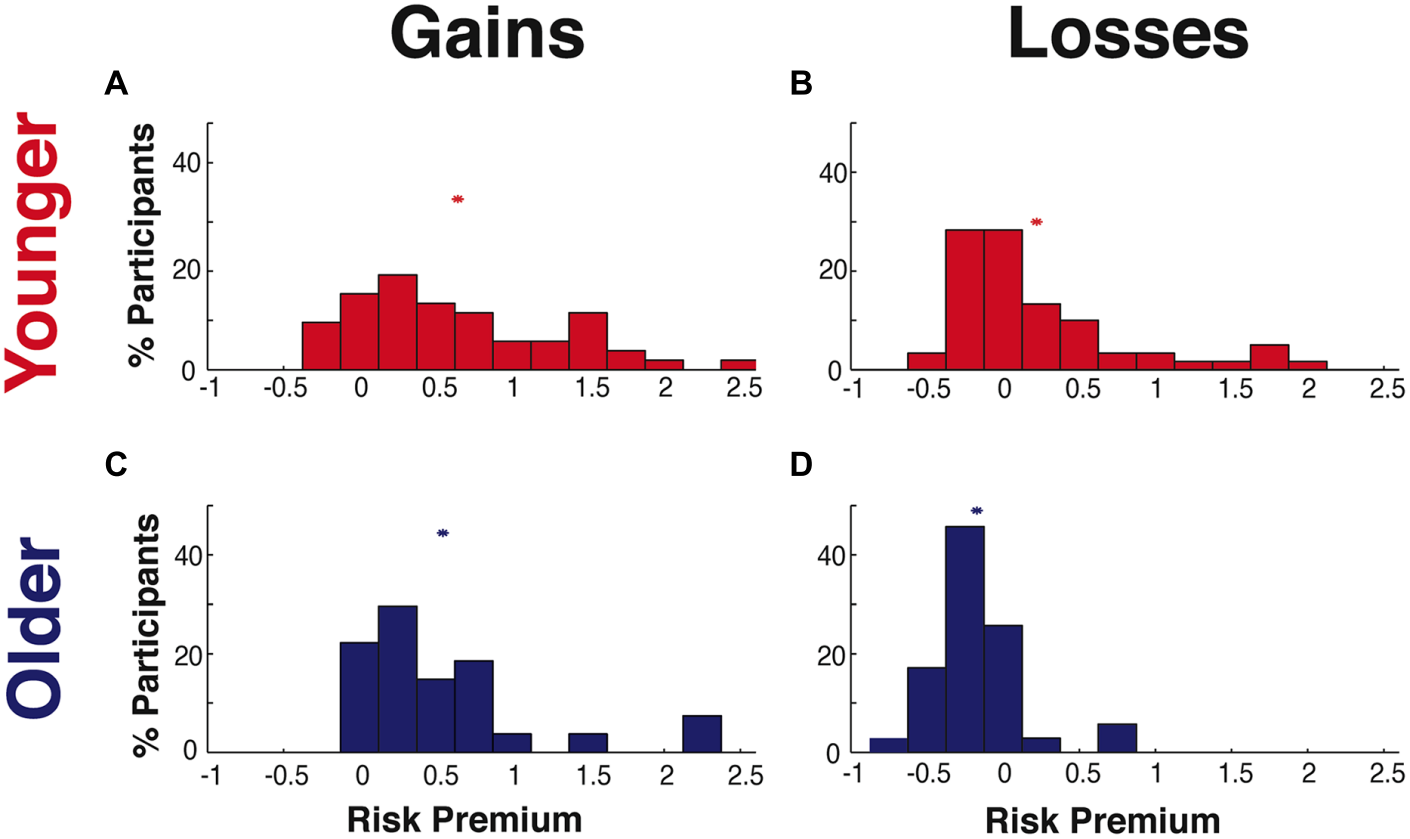
**Risk preferences.** Distribution of individual risk premium values for **(A)** younger adults (YA) in the gains domain, **(B)** YA in the losses domain, **(C)** older adults (OA) in the gains domain, and **(D)** OA in the losses domain. The “^∗^” shows the mean of each distribution.

To examine whether aging alters risk and ambiguity preferences, we contrasted our YA and OA samples, with comparisons listed in **Table 4** and shown in **Figure 3**. Within the gains domain, YA and OA were similarly risk averse [mean SD YA = 0.64 0.66, OA = 0.55 0.61, between group difference *t*(77) < 1, *p* = n.s.]. Within the losses domain, we identified significant age-related differences, with YA risk seeking (mean SD = 0.22 0.59) and OA risk averse [mean SD = -0.17 0.31, between group difference *t*(93) = 3.662, *p* < 0.001].

**Table 4:** Comparison of economic measures between YA and OA.

	YA Mean ± SD	OA Mean ± SD	YA vs. OA *p-*value
***Gains domain***
**Uncertainty premium**
Risk Ambiguity Risk × Ambiguity	0.65 ± 0.66 1.54 ± 1.46 *r*(35) **=** 0.33, ***p* = 0.043**	0.55 ± 0.61 1.46 ± 1.04 *r*(19) **=** 0.55, ***p* = 0.009**	0.52 0.81
**Information strategies**
Choice strategy *r*^2^ rEV *r*^2^ pWIN	0.16 ± 0.24 0.26 ± 0.14 0.10 ± 0.12	0.12 ± 0.22 0.21 ± 0.16 0.09 ± 0.11	0.43 0.15 0.75
**Response time (s)**
Risk Ambiguity	1.55 ± 0.61 1.35 ± 0.52 ***p* = 0.046**	2.49 ± 0.90 2.34 ± 0.83 ***p* = 0.48**	**< 0.0001**
***Losses domain***
**Uncertainty premium**
Risk Ambiguity Risk × Ambiguity	0.22 ± 0.59 0.24 ± 0.78 *r*(56) **=** 0.77, ***p < 0.0001***	-0.17 ± 0.31 -0.18 ± 0.40 *r*(33) **=** 0.68, ***p < 0.0001***	**<0.001** **0.002**
**Information strategies**
Choice strategy *r*^2^ rEV *r*^2^ pWIN	0.38 ± 0.15 0.40 ± 0.13 0.03 ± 0.04	0.31 ± 0.16 0.35 ± 0.13 0.04 ± 0.05	0.052 0.058 0.21
**Response time (s)**
Risk Ambiguity	1.74 ± 0.51 1.69 ± 0.46 ***p* = 0.57**	3.17 ± 1.27 3.37 ± 1.25 ***p* = 0.49**	**< 0.0001**

rEV, relative expected value; pWIN, probability of winning. Overall participants responded slower in the losses tasks than in the gains task with significant difference in OA (*p* < 0.01) and marginally significant difference in YA (*p* = 0.068). Bolded values corresponds to statistically significant test at *p* < 0.05.

A similar pattern of age-related effects was also found for ambiguity preferences (**Table 4**). In the gains domain, participants in both age groups were equally ambiguity averse [mean SD YA = 1.54 1.46, OA = 1.46 1.04, between group difference *t*(60) < 1, n.s.]. While in the losses domain, YA were ambiguity seeking (mean SD = 0.24 0.77) and OA were ambiguity averse [mean SD = -0.19 0.30; *t*(93) = 3.14, *p* = 0.002]. Calculation of Cohen’s *d* indicated moderate to large effect sizes (Cohen, 1988) for age-related differences in both risk and ambiguity preferences within the losses domain (Cohen’s *d*, risk = 0.78, ambiguity = 0.66).

We found correlations between risk and ambiguity preferences within the gains domain [YA: *r*(35) = 0.34, *p* = 0.043; OA: *r*(19) = 0.55, *p* = 0.009], concurring with a recent study (Lauriola and Levin, 2001b). We extend this finding, showing that risk and ambiguity preferences are also correlated within the losses domain [YA: *r*(56) = 0.80, *p* < 0.0001; OA: *r*(33) = 0.68, *p* < 0.0001].

Risk preferences across the gains and losses domains were not significantly correlated within either age group (all |*r*| < 0.08, *p* = n.s.). Similarly, ambiguity preferences across domains were uncorrelated in YA [*r*(35) = -0.11, *p* = n.s.]. However, in OA there was a significant negative correlation between ambiguity preferences across the gains and losses domains [*r*(20) = -0.46, *p* = 0.032]. Given the inverse relationship between the premium metric and preferences across domains (see Quantifying Uncertainty Preferences), this negative correlation shows a positive relationship in OA between ambiguity aversion for gains and for losses.

A potential concern in interpreting the lack of found differences for gains risk preferences between OA and YA could be that highly risk averse participants were ‘cut-off’ by our task design and analyses, which set a ceiling measurable risk premium value of 2.5. This is extremely unlikely, as demonstrated by estimating the likelihood of finding values outside of our measurable range, based upon the observed risk premium values in the remainder of each of our samples and the normal distribution. For YA, the edge is 2.9 SDs from the mean, which indicates that approximately 99.5% of YA should have risk preference values within our measureable range. Similarly, for OA the edge is 3.3 SDs from the mean, indicating that approximately 99.9% of participants should have measurable risk premium values. In other words, based upon the means and variance of our participants with viable risk preference values, we anticipate the presence of fewer than one participant with preferences extreme enough to not fall within our measureable range. We note that while an adaptive task design would avoid this potential concern by fitting trials to individuals, it would also produce additional concerns such as trial order effects.

### Differences in Choice Strategy across the Gains and Losses Domains

**FIGURE 4:**
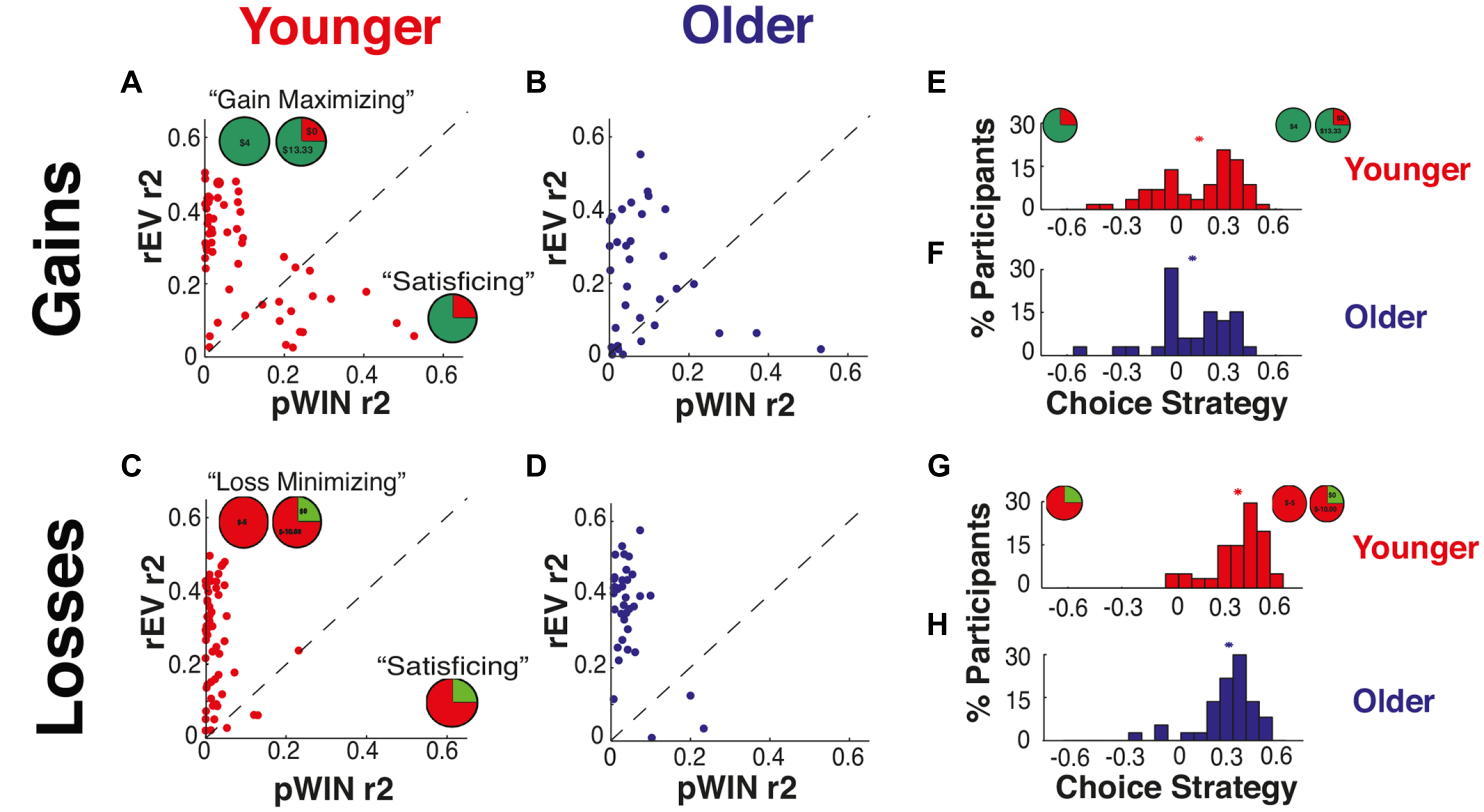
**Choice strategy – utilization of trial information.** Relationship of independent *r*^2^-values of relative expected value (rEV) and probability of winning (pWIN) on trial-by-trial choice behavior for **(A)** YA in the gains domain, **(B)** OA in the gains domain, **(C)** YA in the losses domain, and **(D)** OA in the losses domain. Distributions of choice strategy metric (difference between *r*-squares of rEV and pWIN) for **(E)** YA in the gains domain, **(F)** OA in the gains domain, **(G)** YA in the losses domain, and **(H)** OA in the losses domain. The “^∗^” shows the mean of each distribution.

We examined whether aging altered what information participants relied upon to make their decisions through the use of our choice strategy metric. Choice strategy was determined, within each domain, through linear regressions to determine the maximal influence (expressed through *r*^2^-values) of the rEV and pWIN trial-by-trial information on individual choice behavior. These values were determined separately within each of the gains and losses domains across our YA and OA samples (**Figures 4A–D**).

Within both the YA and OA groups, we observed significantly higher choice strategies in the losses domain than in the gains domain [YA: *t*(117) = 6.00, *p* < 0.0001; OA: *t*(68) = 4.23, *p* < 0.0001] with large effect sizes in both groups (*d*, YA = 1.10, OA = 1.00; **Table 4**; **Figures 4G,H**). As the choice strategy metric is a combination of two factors, we also examine the effects of aging on these factors individually, revealing that the differences were driven by alterations to both components – increased use of the rEV information [YA: *t*(60) = 8.45, *p* < 0.0001, *d* = 1.06; OA: *t*(34) = 5.13, *p* < 0.0001, *d* = 0.94], along with decreased use of the pWIN information [YA: *t*(56) = 4.62, *p* < 0.0001, *d* = 0.82; OA: *t*(32) = 2.23, *p* = 0.033, *d* = 0.64]. A significant correlation between individual choice strategies across the gains and losses domains was present for YA [*r*(55) = 0.42, *p* = 0.001], but absent for OA [*r*(31) = 0.20, *p* = n.s.].

### Effects of Aging on Choice Strategy

Examining for age-related differences in choice strategy, we found no differences within the gains domain [mean SD YA: 0.16 0.24, OA: 0.12 0.22, *t*(89) < 1, n.s.; **Table 4**; **Figures 4E,F**].

Examining for age-related differences within the losses domain, we found that OA exhibited lower choice strategies than YA [mean SD YA: 0.38 0.15, OA: 0.31 0.16, *t*(96) = 1.97, *p* = 0.052, *d* = 0.41; **Figures 4G,H**]. As this change in the composite strategy metric could be driven by either decreased use of rEV information or enhanced use of pWIN information, we examined each component individually. OA showed marginally significant lower use of rEV information [mean SD rEV *r*^2^ values YA: 0.40 0.13, OA: 0.35 0.13, between group difference *t*(97) = 1.92, *p* = 0.058, *d* = 0.40], without alteration in the use of pWIN information [mean SD pWIN *r*^2^ values YA: 0.03 0.04, OA: 0.04 0.04, between group difference *t*(96) = 1.27, *p* = 0.21, *d* = 0.27].

### Relationship between Risk Preference and Choice Strategy within OA

**FIGURE 5:**
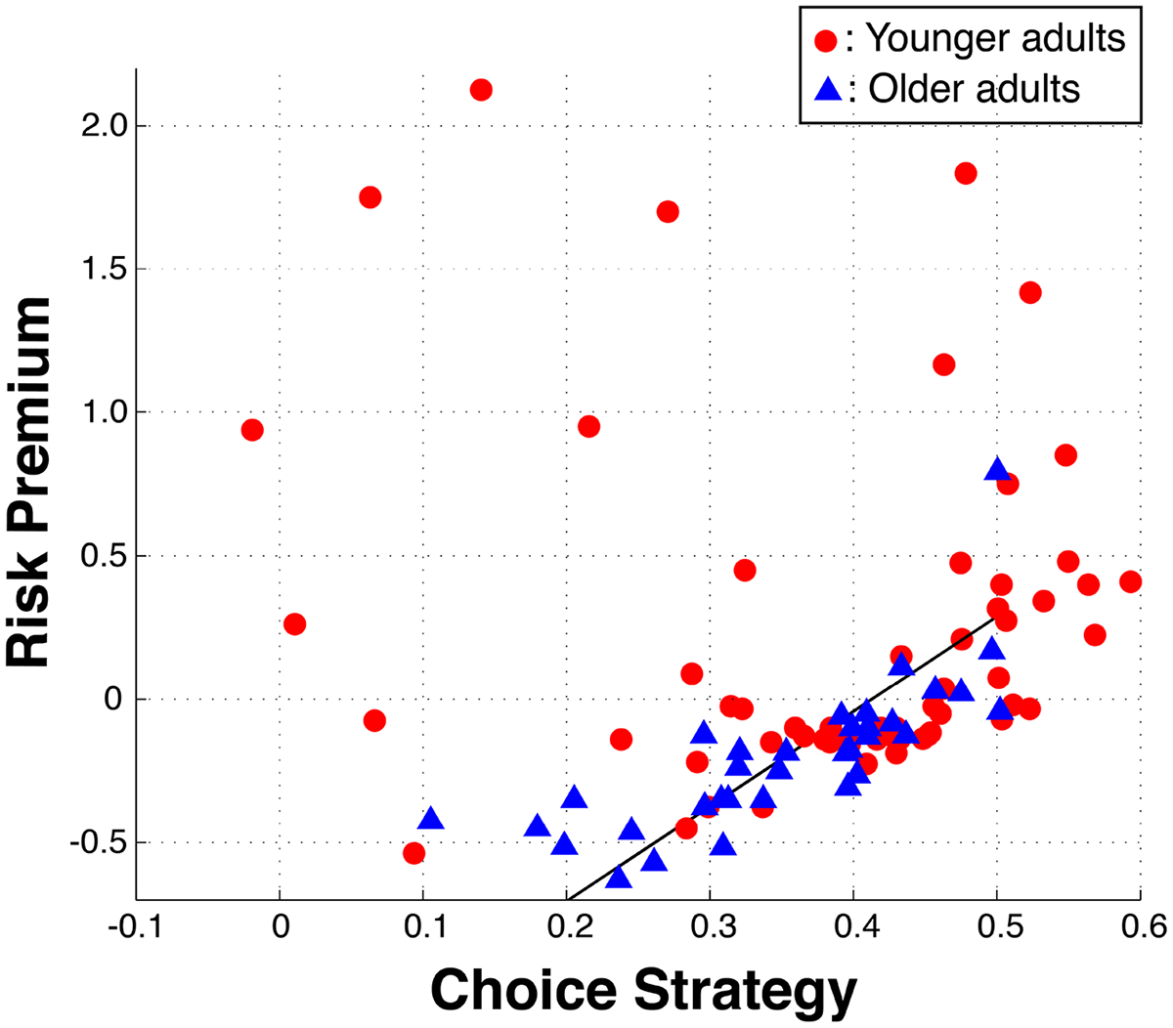
**Interaction between risk preferences and choice strategies in the losses domain.** Within older adults, a positive correlation between risk premium and choice strategy was identified, such that increasing use of the rEV information (maximizing) results in more risk neutral preferences (increased ‘rationality’). The included black line is the total least square line for the older adults.

Given the observed alterations of OA in both risk preferences and choice strategies within the losses domain, we looked for interactions between these metrics (**Figure 5**). We excluded one OA from this analysis, as her risk preference and choice strategy interaction was a strong outlier (>4.95 SD). OA exhibited a highly significant correlation between risk preference and choice strategy in the losses domain [*r*(32) = 0.77, *p* < 0.0001], such that the closer their risk premium was to zero, the higher their choice strategy. In other words, the greater their reliance on the maximizing information, the more risk neutral their risk preference was. This relationship was absent in YA [*r*(57) = -0.11, *p* = n.s.]. Importantly, such a relationship in OA is not due to our task design or metrics, as evidenced by the absence of such a correlation within YA.

## Discussion

We investigated the effects of aging on economic decision-making, focusing on alterations of risk preferences and choice strategies within both the gains and losses domains, contrasting cognitively healthy OA with YA. OA were significantly more risk and ambiguity averse in the losses domain, but were not significantly different from YA within the gains domain. OA also made significantly less use of the maximizing choice strategy in the losses domain. Finally, we found a correlation between risk preference and choice strategy such that the more OA utilized maximizing choice strategies, the more risk neutral (or ‘rational’) their preferences.

### Older Adults are More Risk Averse for Losses

Older adults were significantly more uncertainty averse in the losses domain, but were not significantly different from YA within the gains domain. YA demonstrated the classic pattern of being risk averse for gains and risk seeking for losses (Kahneman and Tversky, 1979). Contrastingly, OA were risk averse across both the gains and losses domains.

Given that OA have less time to recover from financial catastrophe, they are typically advised to shift their retirement savings away from risky investments, (Jagannathan and Kocherlakota, 1996). The preference differences we found between YAs and OAs matches this advice. Our finding also expands upon a study by Ebner et al. (2006), who found OA to be generally oriented towards prevention of losses while YA focused on pursuing gains. Our results suggest that such a change can be extended to the domain of monetary decision making and could be the result of enhanced uncertainty aversion for losses, rather than reduced responses to gains.

It is unclear how such age-related alterations in economic risk preferences may generalize to other domains, such as medical or social decision making (Weber et al., 2002). In fact, while risk aversion may be beneficial in specific circumstances, an overall increase in risk aversion would not be beneficial in all situations. Good decision making is derived from the ability to tailor our preferences to the specific context and goals of the choice.

We note that our risk preference metric, the risk premium, is not the result of a specific theoretical model, but is simply a zero-centered transform of the psychometric indifference point. A potential pitfall of this empirical formulation of risk preference is that it does not ascribe to any specific theoretical model of risk preference, and therefore is not interpretable specifically in-line with those models. However, a potential advantage of such a model-free metric is that it does not rely on specific theoretical assumptions. For example, expected-utility theory states that the power function risk metric is the result of the diminishing weight of marginal utility, but it is unclear if that is a viable mechanism (Rabin, 2000). Similarly, Prospect Theory suggests that the risk preferences of individuals should be highly correlated across gains and losses (reflection effect), but we find no correlation between risk preferences across domains, concurring with other empirical studies (Cohen et al., 1987; Schoemaker, 1990; Laury and Holt, 2000; Tymula et al., 2013). We note, however, the very strong correlations we find between individual risk premium and power function risk preference measures, indicating that these measures do largely account for the same variance across individuals.

### Older Adults have Decreased Maximizing Strategies within the Losses Domain

Within the gains domain, there was no significant difference between the choice strategies of YA and OA. However, within the losses domain, OA showed lower choice strategies than YA, specifically attributable to lower utilization of the calculated rEV information while maintaining equivalent use of the readily available pWIN information as YA.

A possible explanation for why choice strategy was only altered in the losses domain is that participants may have engaged in more effortful cognitive processing within the losses domain, which may have helped reveal age-related differences. The presence of greater effort is backed by the longer response times in the losses domain (**Table 4**), significant in OA and trending in YA. Further, across both YA and OA, we see higher overall choice strategy and specifically increased utilization of maximizing rEV (not just reduced pWIN), suggesting higher motivation in the losses domain than in the gains domain. Such increases in cognitive effort for loss-related decision making concurs with the standard concept of loss aversion, in which people weigh losses more intensely than gains of the same magnitude (Kahneman and Tversky, 1979). High levels of motivation and cognitive effort have been shown to help reveal age-related effects in complex tasks (McDowd and Craik, 1988; Huxhold O et al., 2006). It may be that as aging reduces cognitive capacity, OA adapt by conserving processing resources for highly motivated decisions (Hess et al., 2009). Increased utilization of the maximizing strategy in loss-related decision making may reflect OA consciously choosing to engage in more effortful cognitive processing, but due to limited cognitive resources, OA are unable to match the high performance of YA.

Our finding, that OA made lower use of maximizing information in the losses domain (i.e., lower overall choice strategy metric and specifically decreased rEV), is consistent with prior studies showing that older investors (age 60 and above) are less effective in applying their investment skills due to age-related cognitive decline, even though they have greater investment knowledge and experience than younger investors (Korniotis and Kumar, 2011), although other studies point out that reduced strategy may not necessarily lead to diminished decision quality when simple strategies are viable (Mata et al., 2012).

### Correlation between Risk Preferences and Choice Strategies in OA

Within the losses domain, the OA who utilized the maximizing rEV information, were more risk neutral. In classical economic utility theory (von Neumann and Morgenstern, 1944) rationality is characterized by utility maximization, which translates into consistent use of risk preferences. Within our sample of OA we see a correlation between preferences and strategy, with maximizing strategy driving risk neutral preferences. This pattern is intriguing for three reasons. Firstly, consistent choice behavior is required for high values on the choice strategy metric. As participants show consistent choices over trials, their behavior can be considered more rational. Secondly, OA, as a group, show convergence on a single preference value, driven by the degree to which they utilize the effortful strategy. In an individual, such consistent application of preferences would result in consistent choice behavior and enhanced rational choice. Thirdly, the specific risk preference value that they converge on is risk neutrality, at which utility maximization converges with value maximization. This suggests that the more OA were motivated and engaged in effortful strategies, the more they focused on maximizing the objective value of their choices. In other words, this specific linkage between risk preferences and strategy suggests that OA are exhibiting a relationship between enhanced rationality and enhanced value maximization. Within YA, we see greater variability in the relationship between risk preference and strategy.

One possible explanation for these differences is that OA have acquired experience over their lifetime about not just *what* information to pay attention to (rEV vs. pWIN), but also *how* to utilize that information. Consistent with our findings, a study conducted by Tentori et al. (2001) observed that OA make more ‘rational’ choices (i.e., violations of transitivity while selecting hypothetical supermarket discount cards) than YA, suggesting that age-related accumulation of experience leads to greater rational choice. Such wisdom gained through experiences would then produce our found relationship, with higher motivated engagement in the task (i.e., choice strategy) leading to more neutral preferences.

An intriguing question is whether the effects of aging on economic decision-making are non-linear. Middle-aged adults have been suggested to be better economic decision makers than either YA or OA, at least borrowing at lower interest rates and paying fewer fees (Argawal et al., 2007). Potentially, middle-aged adults could have the highest quality decision making as they have the benefits of acquired life experience without cognitive decline. In addition, further studies are needed to understand how performance on lab-based economic tasks translates to real-world economic behaviors (for example, see Li et al., 2015).

## Conclusion

Understanding the effects of aging on uncertainty preferences and choice strategies has vital implications for OA. Our study investigated the effects of aging on economic decision-making across both the gains and losses domains, specifically examining alterations in uncertainty preferences, choice strategies, and the interactions of the two. We found clear differences in economic decision-making between YA and OA in the losses domain, with no alterations in the gains domain. Within the losses domain, OA were more risk and ambiguity averse and made less use of maximizing choice strategies. Additionally, we identified a positive effect of aging, a correlation between preference and strategy such that the more engaged a participant was (higher choice strategy), the more rational and value maximizing their behavior was. Our results show that healthy aging results in both positive and negative alterations of economic decision-making preferences and strategies.

## Conflict of Interest Statement

The authors declare that the research was conducted in the absence of any commercial or financial relationships that could be construed as a potential conflict of interest.
